# The role of viral particle integrity in the serological assessment of foot-and-mouth disease virus vaccine-induced immunity in swine

**DOI:** 10.1371/journal.pone.0232782

**Published:** 2020-05-05

**Authors:** Florencia Celeste Mansilla, Cecilia Soledad Turco, María Cruz Miraglia, Fernando Aníbal Bessone, Raúl Franco, Mariano Pérez-Filgueira, Juan Manuel Sala, Alejandra Victoria Capozzo

**Affiliations:** 1 IVIT, Instituto de Virología e Innovaciones Tecnológicas, Consejo Nacional de Investigaciones Científicas y Tecnológicas (CONICET), Instituto Nacional de Tecnología Agropecuaria (INTA), Hurlingham, Buenos Aires, Argentina; 2 Estación Experimental Agropecuaria “Marcos Juárez”, INTA, Marcos Juarez, Córdoba, Argentina; 3 Estación Experimental Agropecuaria “Mercedes”, INTA, Merdeces, Corrientes, Argentina; Plum Island Animal Disease Center, UNITED STATES

## Abstract

The efficacy of foot-and-mouth disease virus (FMDV) inactivated vaccines is mainly dependent on the integrity of the whole (146S) viral particles. If the intact capsids disassemble to 12S subunits, antibodies against internal-not protective epitopes, may be induced. Serological correlates with protection may be hampered if antibodies against internal epitopes are measured. Here we compared the performance of different ELISAs with the virus-neutralization test (VNT) that measures antibodies against exposed epitopes. Sera from pigs immunized with one dose of an expired commercial FMDV vaccine were used. This vaccine contained about 50% of O1/Campos and over 90% of A24/Cruzeiro strains total antigen as whole 146S particles. Specific-total antibodies were measured with the standard liquid-phase blocking ELISA (LPBE). We also developed an indirect ELISA (IE) using sucrose gradient purified 146S particles as capture antigen to titrate total antibodies, IgM, IgG1 and IgG2. A good correlation was found between VNT titers and IgG-ELISAs for A24/Cruzeiro, with the lowest correlation coefficient estimated for IgG2 titers. For O1/Campos, however, the presence of antibodies against epitopes different from those of the whole capsid, elicited by the presence of 12S particles in the vaccine, hampered the correlation between LPBE and VNT, which was improved by using purified O1/Campos 146S-particles for the liquid-phase of the LPBE. Interestingly, 146S particles but not 12S were efficiently bound to the ELISA plates, confirming the efficiency of the IE to detect antibodies against exposed epitopes. Our results indicate that any serological test assessing total antibodies or IgG1 against epitopes exposed in intact 146S-particles correlate with the levels of serum neutralizing antibodies in vaccinated pigs, and might potentially replace the VNT, upon validation. We recommend that antigen used for serological assays aimed to measure protective antibodies against FMDV should be controlled to ensure the preservation of 146S viral particles.

## 1. Introduction

Foot-and-mouth disease (FMD) is considered the most economically important disease that affects cloven-hoofed animals such as pigs, cattle, sheep and goats [1]. It is caused by a picornavirus, the foot-and-mouth disease virus (FMDV), which comprises 7 serotypes and numerous subtypes. FMD is enzootic in large regions of the world [2], especially in Asia, Africa and, to a lesser extent, South America, where vaccination is used as a preventive method. Currently, commercially available vaccines are based on chemically inactivated whole viral particles that are formulated with aqueous or oil adjuvants [3].

Pigs are highly susceptible to oral infection with FMDV, presenting higher severity than ruminants [4]. Pigs serve as airborne amplifiers of FMDV because one infected pig can excrete up to 3,000 times more viral particles per day that a sheep or a cow [5]. Given the importance of the pig in the transmission of foot-and-mouth disease and the current context of pig industry growth worldwide, there is a strong need for simple and high-performance serological techniques applicable to epidemiological monitoring and vaccine efficacy studies for this specie. Currently, the virus neutralization test (VNT) is applied. This assay is difficult to standardize, cumbersome and inadequate to be used on a large scale. Moreover, it involves the manipulation of live virus, which results in the risk of an outbreak. This point is particularly relevant for FMDV-free regions, where live virus can only be manipulated under strict biosafety conditions. That is the reason why ELISAs are preferred, as they use inactivated virus, are high-throughput and easily deployable to any laboratory [6].

Total antibodies are usually assessed by Liquid-Phase Blocking ELISA (LPBE), which requires an inactivated virus suspension as well as capture and detector antibodies that are usually prepared by immunizing rabbits and guinea pigs. These assays must be set-up for each vaccine strain, consequently, they are useful for vaccine potency testing, but they are not convenient in the case of an outbreak with a non-related strain, since capture and detector antibodies need to be produced and standardized. Measuring total IgG titers by ELISA does not provide any information concerning the functionality of antibodies, and this is thought to be the reason why a low correlation is found between LPBE titers and VNT or protection [6], which may explain why the use of ELISAs is limited. There is a need for well-defined markers for immunity induced by FMD vaccination. These markers could serve as surrogates of vaccine protective efficacy and would be helpful for the quick introduction of new or improved vaccines in the future [5]

The infective virus particle has a sedimentation coefficient of 146S. It contains one molecule of single stranded positive sense viral RNA and protein capsid comprising 60 copies of proteins called VP1, VP2, VP3 and VP4, which is located internally. Under certain conditions, 146S particles can undergo an irreversible dissociation into stable pentamers (12S) that lack VP4 [7–9]. Neutralizing antibody production is associated mainly with 146S particles [10] and infectivity-related sites are exposed on the FMDV particle [11] whereas 12S particles stimulate the production of low levels of neutralizing antibodies. Even when both the 146S and 12S particles contain the same proteins, the conformation of the polypeptides is different in the two particles and the configuration required for the production of neutralizing antibody is largely lost when the 146S particles are converted into 12S particles, as demonstrated before [17]. Moreover, vaccine efficacy relies on the presence of complete-intact viral particles [12,13], mainly due to the capacity of this antigen to elicit antibodies against exposed epitopes, which can conduct viral clearance in the case of infection.

Intact viral particles can be differentiated from pentamers by means of sucrose gradient ultracentrifugation or size-exclusion chromatography [12–14]. Capsid integrity may be affected by different vaccine excipients [15], thiomersal [16], mild temperatures and pH variations even if they occur for a short period, and particle stability is strongly dependent on the viral strain [17,18]. For instance, A-strains´ capsids remain intact after an overnight incubation at 37°C while their O-strains counterparts do not [17]. Therefore, a long inactivation step may disrupt O-strain capsids, and this fact has never been considered when using FMD antigen for serology.

Even though the need of antibodies against exposed-neutralizing epitopes elicited by intact capsids is well accepted as a correlate of protection by the FMD community [13,19], the role of 146S capsid stability in serology has never been assessed. In addition, vaccine-induced antibody response in terms of kinetics of neutralizing antibodies, total antibodies, and IgG subtypes has not been deeply characterized in pigs. The aim of this work was to develop serological techniques to assess the different aspects and kinetics of the antibody response against FMDV in vaccinated pigs using purified viral particles that can be prepared from any field virus. We hypothesized that the lack of concordance between VNTs and ELISA titers may be due to the measurement of antibodies against internal “non-protective” epitopes that bind to disrupted particles present in the antigen preparation used for ELISA. In this regard, the involvement of antigen stability both in the vaccine and the ELISAs were considered and analyzed in terms of correlation with the neutralizing antibody titers.

## 2. Materials and methods

### 2.1 Animals

A group of 15 two-months-old piglets born to naïve dams, which had been recently weaned, were used (mean weight: 10Kg, Canadian genetics). They were housed in a dedicated facility at the Marcos Juarez Agricultural Experimental Station (INTA) located in Córdoba province, Argentina. The sheds had enough space for 15 animals and *ad-libitum* water supply, feeder and cleaning grid. Animal handling and sampling was carried out on this site, under the supervision of INTA´s veterinarians and following the guidelines of the local animal welfare committee. The national authority for animal health (SENASA) approved the procedures (permit letter dated July 15^th^, 2015). Piglets were negative for antibodies against non-structural proteins at the beginning of the experiments (tested with the Priocheck FMDV-NS kit, ThermoFisher Scientific, MA, USA).

### 2.2 Vaccination and sampling

A commercial vaccine was used. The formulation applied in Argentina is a single-oil emulsion containing four inactivated FMDV strains: O1/Campos/Brazil/58 (O1/Campos), A24 Cruzeiro/Brazil/55 (A24/Cruzeiro), A/Argentina/2001 (A/Arg/01) and C3/Indaial/Brazil/71 (C3/Indaial) [20]. The vaccine was applied 22 months after the expiration date. The content of 146S and 12S particles was estimated from the areas under the curves after reading the sucrose gradients (see below) with a serotype-specific antigen ELISA, as described before [17].

Twelve animals were immunized with 2 mL of vaccine by the intramuscular route in the neck, following the producer´s specifications for pigs. Three animals were left unvaccinated as sentinels. Serum samples were taken at 0, 10, 21- and 60-days post-vaccination (dpv).

### 2.3 Virus purification

FMDV A24/Cruzeiro and O1/Campos strains were kindly provided by Biogénesis-Bagó S.A. (Argentina) as inactivated and concentrated preparations from clarified-infected cell cultures. Purified particles were obtained by a 15%-45% sucrose density gradient (SDG) centrifugation method [21] further optimized in our laboratory [22]. Aliquots were kept at -80°C. For estimating the percentage of 146S and 12S particles in the antigen phase, 10mL of the vaccines was treated overnight with one volume of chloroform and the extracted aqueous fraction was analyzed in a sucrose gradient. Collected fractions were analyzed by antigen ELISA following standard methods [23].

### 2.4 Neutralization assay

Serum neutralizing antibodies were titrated by a conventional virus neutralization test (VNT) using infective culture-adapted FMDV O1/Campos or A24/Cruzeiro strains (titer 10^7^ TCID50/ml) on BHK-cell monolayers as described in the OIE Manual of Diagnostic Tests and Vaccines for Terrestrial Animals [24] and adapted by Bucafusco et al [25]. The endpoint titers of the serum samples were expressed as the logarithm (base 10) of the reciprocal of the last dilution of serum that neutralized 100× TCID50 of the virus in 50% of the wells.

### 2.5 Liquid-phase blocking ELISA

Total anti-FMDV O1/Campos and A24/Cruzeiro antibodies were assessed by LPBE performed as stated by the OIE Manual [24] using serotype-specific rabbit serum as capture antibody [25]. Serum samples were incubated with a standardized dilution of a preparation of inactivated virus preserved in glycerol and free (unbound) virus was detected with a strain-specific guinea-pig antiserum. Alternatively, sucrose-gradient purified 146S particles were used as ELISA antigen. Antibody titers were expressed as the reciprocal of the logarithm (base 10) of the last dilution of serum giving the 50% of the absorbance recorded in the virus control wells without serum [26].

### 2.6 Total antibody indirect ELISA

Assessment of specific IgG was performed as described by Lavoria et al. [27] with the following modifications. Inactivated O1/Campos or A24/Cruzeiro 146S purified particles were used as capture antigen in carbonate-bicarbonate buffer (pH = 9.6), by incubating overnight at 4°C. After blocking for 90 minutes (PBS/ 10% Equine serum), different serum dilutions (from 1: 25 to 1: 12,800) were tested and the presence of anti-FMDV antibodies was revealed with a peroxidase-conjugated goat anti-swine IgG diluted 1:3000 (BioRad, CA, USA) followed by ABTS/H_2_O_2_. Titers were estimated as the reciprocal of the dilution giving an OD value above the cut-off, estimated as the average OD value of pre-immune sera diluted 1:25 + 2 SD.

We estimated the intra-plate and intra-day repeatability of this indirect ELISAs. The intra-laboratory repeatability (intermediate precision) was estimated with results obtained by two different technicians, using 3 replicates of ten samples with high VNT titers against O1/Campos strain (VNT titer >1.7). Samples were run in three different days, using three different reagent batches. Coefficients of variation for positive control samples were below 10% for all the operators for each assay. No significant differences were found between mean OD values determined by two different laboratories (p >0.05). ANOVA analysis showed there were no differences in the OD values due to the operator or their repeatability (p values were 0.55 and 0.68 respectively), meaning that the assays can be performed on different days and by different operators without modifying the results.

### 2.7 Isotype ELISAs

The procedure and set-up for IgM, IgG1 and IgG2 ELISAs were the same as described for total IgG except that different conjugates were used. Mouse anti-swine IgG1 (MCA635GA, clone K139 3C8, 1:7500), IgG2 (MCA636GA, clone K68 Ig2, 1:10000) and IgM (MCA637GA, clone K52 1C3 1:10000) were used (all from BioRad), followed by a goat anti-mouse IgG peroxidase conjugated (1:1000, KPL, MD, USA), adapted from Capozzo et al 1997 [28] and Lavoria et al. 2012 [27].

### 2.8 Binding capacity of 146S vs 12S articles to ELISA plates

A preparation containing 1,300 μg/mL of whole viral purified particles of O1/Campos, preserved in aliquots at -80°C, was either used directly to coat plates with PBS or carbonate/Bicarbonate buffer (pH: 9.6) or treated at 60°C for 20 minutes to produce 12S particles, as described before [17]. Different ratio of 146S to 12S particles were prepared and used to coat duplicate wells. The absence of 146S particles in the heated preparations was confirmed by sucrose-gradient ultracentrifugation. Purified IgG at the same concentration was used as control. ELISA plates were incubated overnight at 4°C, following the standard procedure. The next day, plates were washed five times with PBS-Tween 20 (0.05%) and bound total proteins were quantified using Micro BCA kit (Thermo Fisher) following the manufacturer´s instructions.

The same design was used to run 10 serum samples from 60 dpv vaccinated animals (diluted 1: 50) comparing the OD values for each captured antigen preparation with that of the whole particles (expressed as percentage of residual reactivity)

### 2.9 Statistical analysis

Area under the curve was estimated using the trapezoid rule. Serological assays were performed blinded to the results of the other tests and were then compared all together and to their VNT titers. VNT titers were used to build scatter plots showing the relationship between values produced by the different ELISAs and VNT titers. To determine the level of agreement between results obtained by the different ELISAs and VNT we computed Pearson´s correlation and linear regression analysis. Pairwise comparisons were assessed by the Mann-Whitney test, and results from multiple treatments were compared by means of the Krustall-Wallis test, followed by the Dunn´s multiple-comparisons test. In all instances, a confidence interval of 95% was considered. The analyses were performed with GraphPad Prism v5.0 (GraphPad Software, CA, USA).

## 3. Results

### 3.1 Kinetics of Ig-subtype responses in vaccinated pigs

**Fig 1:**
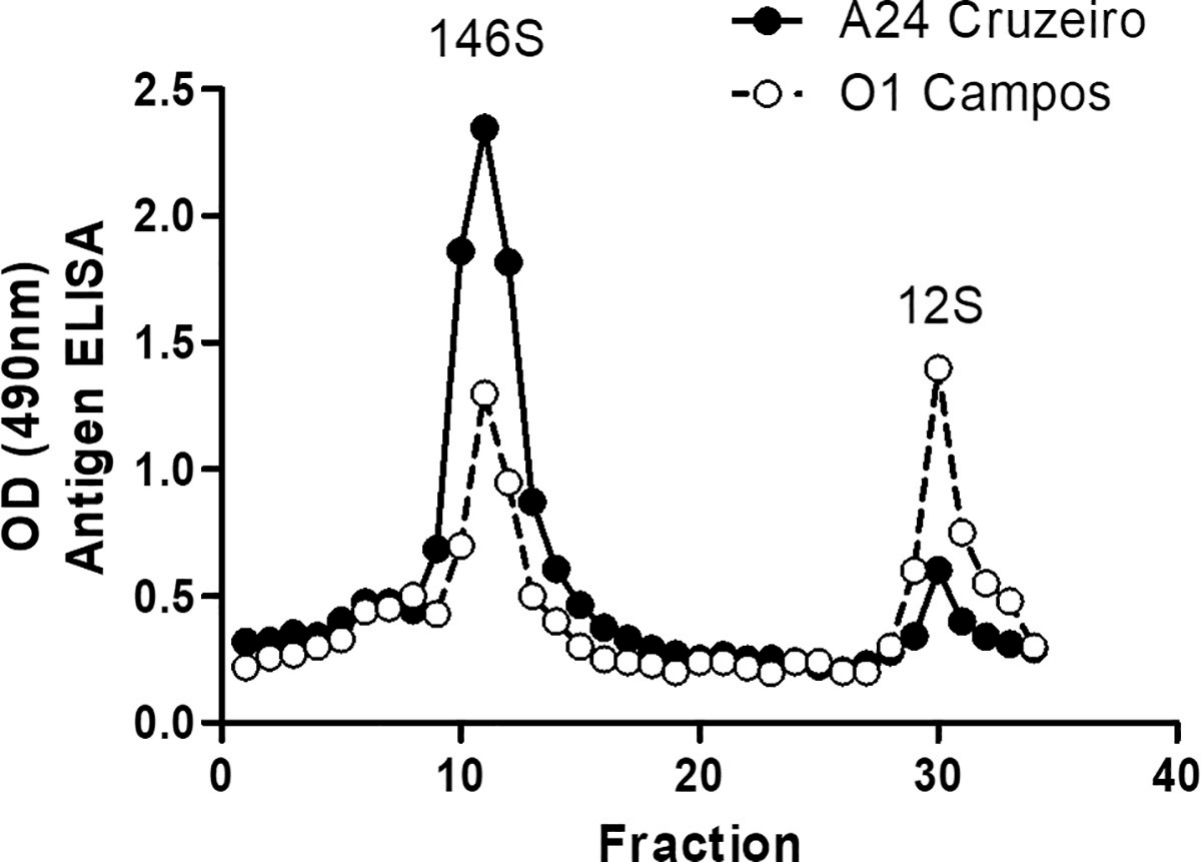
Antigen content in the expired FMDV vaccine. The aqueous phase was extracted with one volume of chloroform and run in a sucrose gradient. Fractions were recovered, read by OD = 260 and then antigen peaks were identified with a strain-specific antigen ELISA. Peaks for 146S and 12S particles are indicated.

An expired commercial vaccine was used and the relative content of whole and disrupted particles was estimated by sucrose gradient ultracentrifugation. Two peaks were identified by reading the collected gradient fractions in a strain-specific antigen ELISA (Fig 1), corresponding to 146S (peak 1) and 12S particles (peak 2). Relative content of both antigenic species was estimated by computing the area under the curve (Fig 1). The applied vaccine had approximately 93% of intact 146S A24/Cruzeiro particles and ~50% of the total amount antigen were whole O1/Campos particles (Table 1).

**Table 1:** Analysis of the area under the curve. Data from Fig 1.

	A24 Cruzeiro	O1 Campos
Baseline	0,300	0,300
Total Area	7,473	5,599
Total Peak Area	6,959	4,695
Number of Peaks	2,000	2,000
Peak 1 (146 S)		
First X =	9,000	9,000
Last X =	17,80	15,00
Peak X =	11,00	11,00
Peak Y =	2,346	1,300
Peak Y—Baseline =	2,046	1,000
Area =	6,469	2,415
% Area =	92,95	51,44
Peak 2 (12S)		
First X =	28,32	28,00
Last X =	33,57	34,00
Peak X =	30,00	30,00
Peak Y =	0,6040	1,400
Peak Y—Baseline =	0,3040	1,100
Area =	0,4906	2,280
% Area =	7,050	48,56

**Fig 2:**
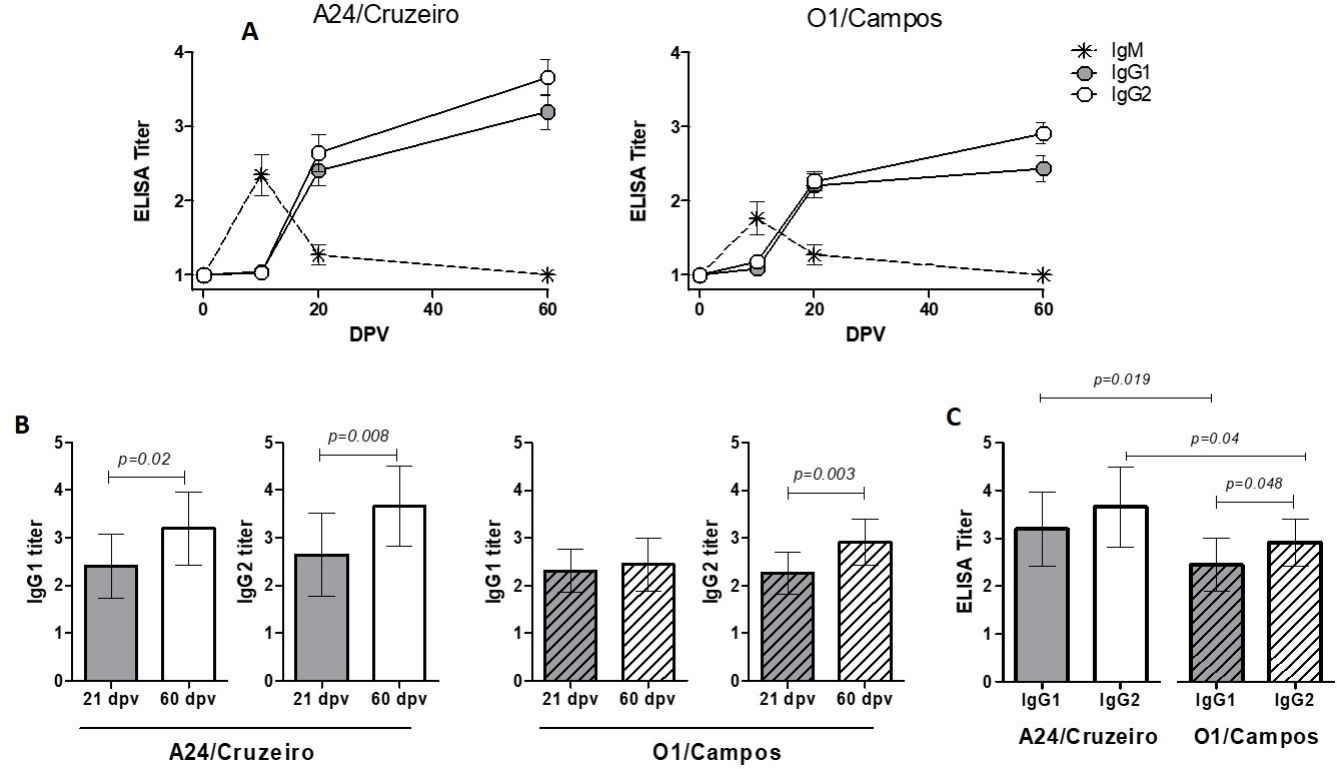
Isotype responses. (A) Kinetics of IgM, IgG1 and IgG2 in vaccinated pigs, mean titers ± SD are depicted. (B) Comparison of mean IgG1 and IgG2 titers at 21 and 60 dpv against both A24/Cruzeiro and O1/Campos strains. (C) Pairwise comparative analysis of IgG1 and IgG2 levels measured at 60 dpv.

A group of 12 piglets was immunized with this vaccine, and three animals were left unvaccinated. Serum samples collected at 0, 10, 21 and 60 dpv were assessed for neutralizing, total antibodies, IgM, IgG1 and IgG2 titers. Vaccinated pigs elicited IgM against both strains shortly after vaccination, high levels were detected at 10 dpv. At that time point, IgG was undetectable in most of the animals. IgM levels decayed by 21 dpv and the IgG switch occurred with a concomitant increase of both IgG1 and IgG2 titers (Fig 2A). Kinetics of IgG1 and IgG2 responses were different for both strains (Fig 2B). While IgG2 levels increased for both strains between 21 and 60 dpv, IgG1 titers remained within the same levels at both time points for O1/Campos and increased significantly for A24/Cruzeiro (Fig 2B). At 60 dpv, IgG2 titers were higher than IgG1 against O1/Campos (p<0.05; Fig 2C) for O1/Campos. Both IgG1 and IgG2 titers were higher for A24/Cruzeiro than for O1/Campos at 60 dpv (Fig 2C).

### 3.2 Total and neutralizing antibodies induced after vaccination

**Fig 3:**
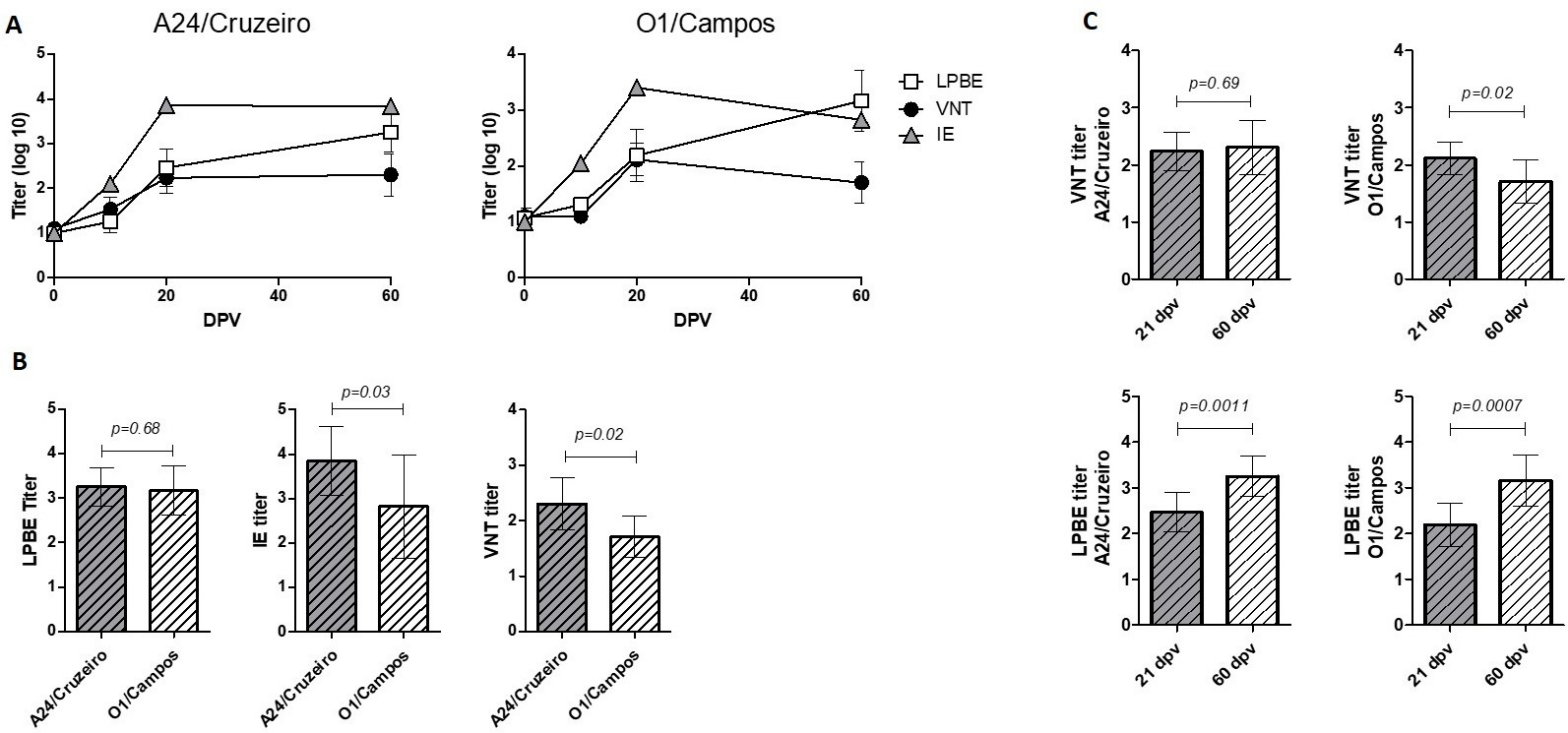
Total and neutralizing antibodies. (A) Kinetics of neutralizing antibodies (VNT) and total antibody titers measured with liquid-phase blocking ELISA (LPBE) or indirect ELISA (IE) as indicated. Mean titers ± SD are depicted. (B) Comparison of LPBE, IE and VNT titers at 60 dpv against A24/Cruzeiro and O1/Campos strains. (C) Pairwise comparative analysis of neutralizing and total antibody levels (estimated using LPBE) at 21 vs 60 dpv, for both strains. Statistically significant differences between groups are indicated.

Serum samples were assessed for neutralizing and total antibodies using the VNT and two different ELISA assays, LBPE and IE. Total antibodies were detected as soon as 10 dpv when using IE while only some animals were positive by LPBE. Antibody titers increased significantly from 10 to 21 dpv (p<0.05) for both virus strains and had similar levels until the end of the experiment (Fig 3A). Neutralizing antibody titers against A24/Cruzeiro were above the detection levels for some animals at 10 dpv while none of the animals elicited detectable neutralizing antibodies against O1/Campos at this time point (Fig 3A). VNT titers increased for both viruses at 21 dpv (Fig 3A) with mean values significantly higher than pre-immune titers (p<0.05).

At 60 dpv, total antibodies measured by LPBE were similar between both strains, while IE and VNT showed differences, with significantly lower titers for O1 Campos compared to A24/Cruzerio strain (p<0.05; Fig 3B).

We then compare the raise in total and neutralizing antibody titers between 21 and 60 dpv. VNT titers were similar between 21 and 60 dpv for A24/Cruzeiro but significantly different for O1/Campos strain (p<0.05; Fig 3C). The same trend was found for IE titers while LPBE levels remained invariable between both time points (Fig 3D).

### 3.3 Correlation between ELISA and VNT titers

**Fig 4:**
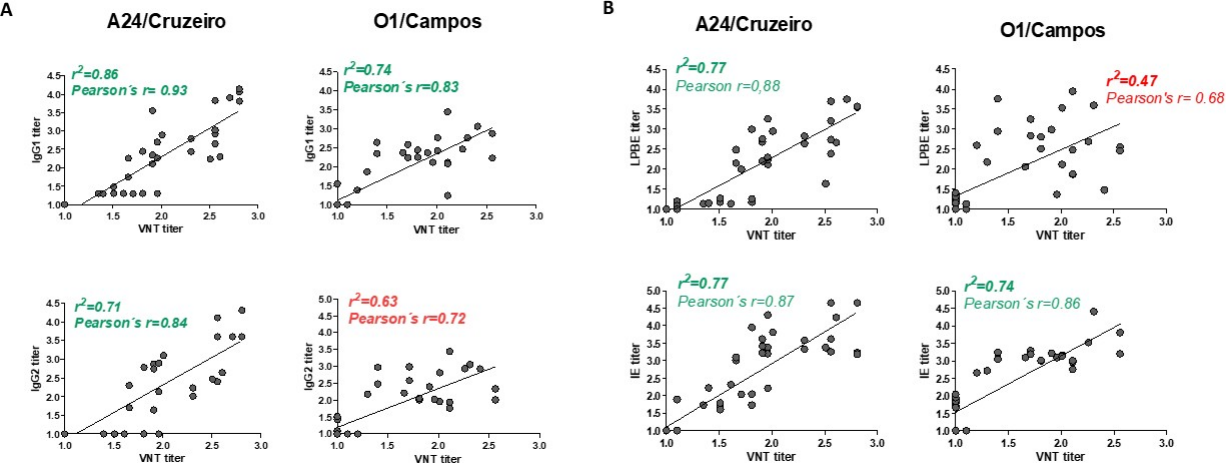
Correlation between virus neutralizing and ELISA titers. (A) Correlation between neutralizing antibody titers (VNT titer) and IgG1 (upper panel) or IgG1 (lower panel) for both A24/Cruzeiro and O1/Campos strains. (B) Correlation analysis between VNT titers and total antibody levels estimated by LPBE (upper panels) or IE (lower panels). Linear correlation coefficients (r2) and Pearson´s r values are shown in each graph. Red values indicate a poor correlation between VNT and IgG2 or LPBE titers for O1/Campos strain.

Correlation analyzes were performed between neutralizing antibody titers and both IgG-subtype and total antibody levels measured by ELISA (Fig 4). Correlations were significant for all the ELISAs when measuring antibodies against A24/Cruzeiro, with the highest correlation coefficient for IgG1 (r^2^ = 0.86), followed by LPBE and VNT (r^2^ = 0.77), being the lowest (r^2^ = 0.71) for IgG2 titers (Fig 4A). Correlation analysis for O1/Campos titers yielded different results. While there was a good correlation between both IgG1 and IE titers (Fig 4B) and VNT (r^2^ = 0.74), coefficients were much lower for LPBE and IgG2 levels (r^2^ = 0.47 and r^2^ = 0.63, respectively).

### 3.4 Relevance of whole particles in FMDV serological assessments

**Fig 5:**
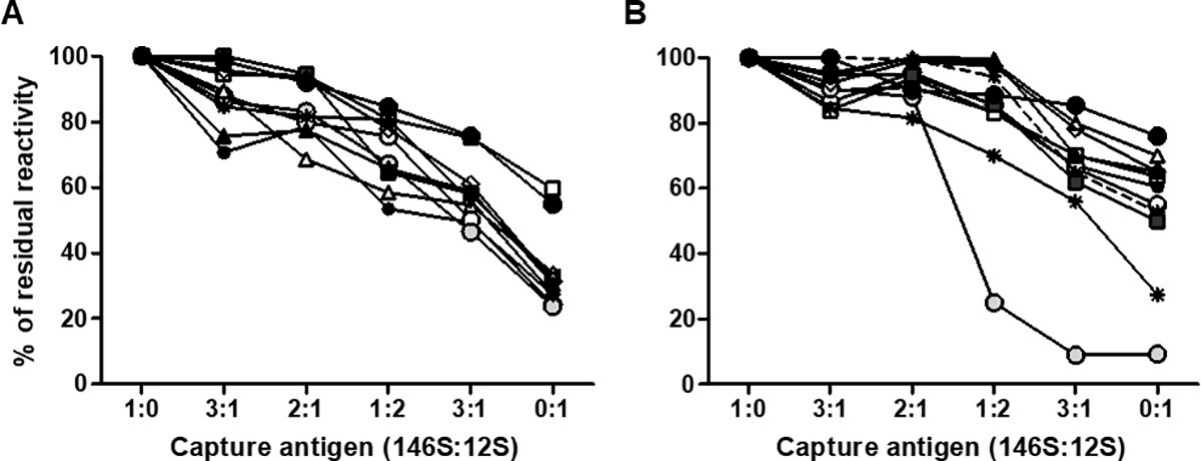
Impact of binding 12S particles in indirect ELISA. ELISA plates were coated with different amounts of 146 S and 12S particles of O1/Campos (A) or A24/Cruzeiro (B), mixed together at the indicated rates. Serum samples from vaccinated pigs were measured in single dilution (1:50) and OD values were compared to those obtained for whole 146S particles and expressed as percentage of reactivity.

Antibody levels induced against each one of the FMDV strains included in current commercial vaccines are always similar and comparable. However, as it was shown previously, the ratio between whole and disrupted particles in the expired vaccine was different for the O1/Campos and A24/Cruzeiro strains. As there was an evident effect of the relevance of measuring antibodies against whole capsids by IE and the correlation of these antibodies against exposed epitopes and VNT titers, we designed an experiment to assess if the increased amounts of 12S pentamers bound to the ELISA plate could modify the correlation with VNT. ELISA plates were coated with different ratios of 146S to 12S particles from the O1/Campos and A24/Cruzeiro strains, tested with a single dilution (1:50) of 60 dpv serum samples, and results were compared to those estimated by using only purified146S particles (Fig 5).

Serum reactivity against the coating antigen diminished when increasing the amount of 12S particles in the coating mixture, this was a consistent result yielded by each tested sample. This means that either antibodies against capsid-internal epitopes exposed in 12S particles are absent or that 12S particles were not efficiently bound to the plate. To test this later hypothesis, we coated plates with an excess amount of either 146S or 12S particles and then quantified total protein effectively bound to the plate. Two different coating buffers were tested in order to see if there was a role of the buffer in the binding capacity of the viral particles to the ELISA plate. Results are shown in Table 2. While 146S particles reached the maximum binding capacity of the wells, 12S particles bound less efficiently. Moreover, the use of carbonate/bicarbonate buffer precluded the binding of 12S pentamers, and only 20% of total protein was retained onto the ELISA plate, meaning that the IE coating conditions will favor the binding of whole viral particles.

**Table 2:** Binding of 146S and 12S particles onto the ELISA plates. Excess amount of antigen was coated ON using either PBS or Carbonate/bicarbonate buffer. Plates were washed after the ON incubation at 4°C and total coated proteins were quantified using a commercial kit.

Antigen	PBS	Carb/Bicarb
146S	14.48	13.77
12S	6.36	3.07
Purified IgG	14.59	15.01

**Fig 6:**
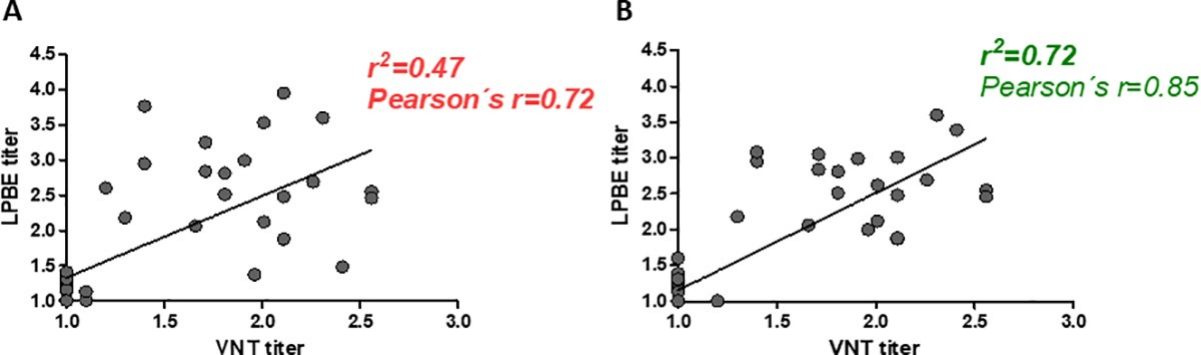
Impact of binding the presence of 12S particles in the correlation with VNT titers. LPBE was performed either with a virus suspension (A) or whole-purified 146S particles (B). Titers were computed and plotted against their corresponding neutralizing titers. Each point depicts an individual sample. Linear correlation coefficients (r2) and Pearson´s r values are shown in each graph. Values in red depict a poor correlation between VNT and LPBE titers.

As a whole, these results would indicate that good correlation with VNT titers required the measurement of antibodies against exposed epitopes present in whole viral particle. If this is correct, then the correlation between LPBE and VNT titers should be improved if performing the assay (specifically the incubation of the virus with serum antibodies in a liquid phase) in the absence of 12S particles. To test this hypothesis, we designed an experiment using freshly purified 146S O1/Campos particles for the liquid phase, incubating at 4°C for two hours (instead of 1h at 37°C) to ensure the preservation of the whole viral particles. We verified that performing LPBE with 146S particles improved the correlation with VNT, modifying the correlation coefficient from 0.47 with the regular un-controlled antigen to 0.72 when using exclusively intact viral particles (Fig 6).

## 4. Discussion

The aim of this work was to assess humoral responses in FMDV vaccinated pigs, considering different aspects of the antibody response apart from neutralizing antibodies, such as total antibodies and isotypes induced. For this purpose, we developed indirect ELISA assays adapting those already used for bovine sera in our laboratory [27].

A well-known fact among FMDV laboratories is that titers estimated by the currently used ELISAs do not always correlate with those measured by the VNT. Others and we have proposed that the lack of correlation is a consequence of not measuring the correct protection marker [6,29]. Here we demonstrated that, besides that, a simple technical issue like using disassembled viral particles may account, at least partially, to the low correlation sometimes observed; and that this phenomenon can be especially important for certain labile strains.

It is well known and demonstrated by our group and others, that type-O are less stable than A strains [17]. In addition, different authors have demonstrated that disrupted viral particles (12S pentamers) are less immunogenic and elicit a diminished neutralizing response [13,19]. Moreover, 12S particles may expose internal epitopes to B-cells, and antibodies against non-protective epitopes can be elicited. In order to analyze the immune response in these conditions we used an expired vaccine that contained only half the amount of the total O1 Campos-antigen as 146S particles. In contrast, due to the intrinsic stability features of A and O strains, most of the whole A24/Cruzeiro particles were preserved.

Isotype responses as well as total antibody titers against commercial FMD vaccines have not been deeply characterized in pigs. A typical primary immune response was observed, characterized by a peak of IgM at 14 dpv that decreased at 21 dpv, as previously shown by Ouldridge et al and Cox [30,31] et al. IgG responses were detected in some animals at 14 dpv and in all of them at 21 dpv. Cox et al showed that IgG switch can occur from 9 dpv [31], with an IgG1 peak around 28 days, followed later by IgG2. Here we found that both IgG1 and IgG2 increased significantly between 21 and 60 dpv for both strains. At 60 dpv, IgG1 and IgG2 titers against A24/Cruzeiro were similar, while significant differences between these isotypes were observed at 60 dpv for O1/Campos. It is important to consider differences related to the assay itself, as Cox et al. used a sandwich ELISA with a capture hyper-immune serum that can potentially bind both whole and disrupted particles. We speculate this difference may be related to the presence of antibodies against internal, protection-irrelevant epitopes. In this regard, it is worth noting that IgG2 titers correlate poorly with VNT titers.

Several studies described the kinetics of FMDV-neutralizing responses using commercial vaccines [32]. VNT titers have been associated with protection in pigs. A study described high levels of protection with VNT titers over 1.7 in pigs against an O strain and claimed that antibody titers between 1 and 1.6 are in a so-called “grey zone” where protection cannot be discerned [33]. In our study, the vaccine induced antibody levels over 1.6 at 21 dpv. These peak levels were maintained at 60 dpv for A24/Cruzeiro, but not for O1/Campos. The high content of 12S particles in O1/Campos vaccine might not favor the adequate development of T-cell immunity, known to require whole 146S-particles to be tackled [17]. In this regard, it is important to consider the differences in the immunogenicity among virus strains (i.e. due to their intrinsic particle stability) that can also impact in the antibody repertoire and avidity maturation.

Indirect ELISAs use sucrose-gradient purified 146S particles as coating material, and samples are incubated directly onto this solid-phase, while LPBE involves an incubation step of the sample with an inactivated virus-culture suspension. Protocols for BEI inactivation of the infectious FMDV supernatants in the serology laboratories are usually the same for any strain, with a fixed amount of BEI concentration, time and temperature. These conditions may be harsh from some strains and compromise the stability of the whole capsids, affecting the final readout of the diagnostics. This is an important issue when evaluating vaccine efficacy using serology as antibodies against non-exposed epitopes, elicited by 12S particles included in the vaccines, may be detected but are irrelevant for protection.

The virus neutralization test is by itself a selective assay that only measure antibodies against exposed epitopes. The use of intact viral particles to coat plates together with the fact that 12S pentamers are not bound to the plate, position indirect ELISAs as a more reliable option than blocking or liquid phase ELISAs for measuring protective antibodies. An additional advantage of IE is avoiding the need of using a capture or detector antibody, which may not be timely available for an emerging field strain.

Finally, a hear-and-say issue around binding purified particles directly to the ELISA plates is the possibility of losing structure once the virus is coated. The good correlation observed with VNT titers by all the assays based on purified virus, irrespectively of what they measure, together with the fact that this good correlation is achieved even when using serum samples with antibodies against internal epitopes, are strong evidences of the conservation of the particle structure once bound to the ELISA plates.

We believe that an important contribution of this study is the set-up and evaluation of an indirect ELISA based on purified-146S particles as coating antigen for titrating total antibodies in swine sera. This assay gives a better correlation with VNT and avoids the need of developing capture and detector antibodies. The use of this ELISA should be considered as an alternative to the VNT provided a fit-for-purpose validation is conducted, probably also reducing cross-reactivity between strains. In conclusion, we strongly recommend that virus used for serological assays should be controlled for capsid integrity to ensure the detection of antibodies against exposed (protection-relevant) epitopes.

## Supporting information

S1 Fig Control of particle stability before performing ELISA (example for O1/Campos, experiment shown in Fig 6). The dotted square indicates the fractions used in ELISA.
(TIF)
