# Supplementary figures and images for: The role of viral particle integrity in the serological assessment of foot-and-mouth disease virus vaccine-induced immunity in swine
Source: PLoS One. 2020 May 5;15(5):e0232782. doi: 10.1371/journal.pone.0232782 (PMC7199947; doi:10.1371/journal.pone.0232782)

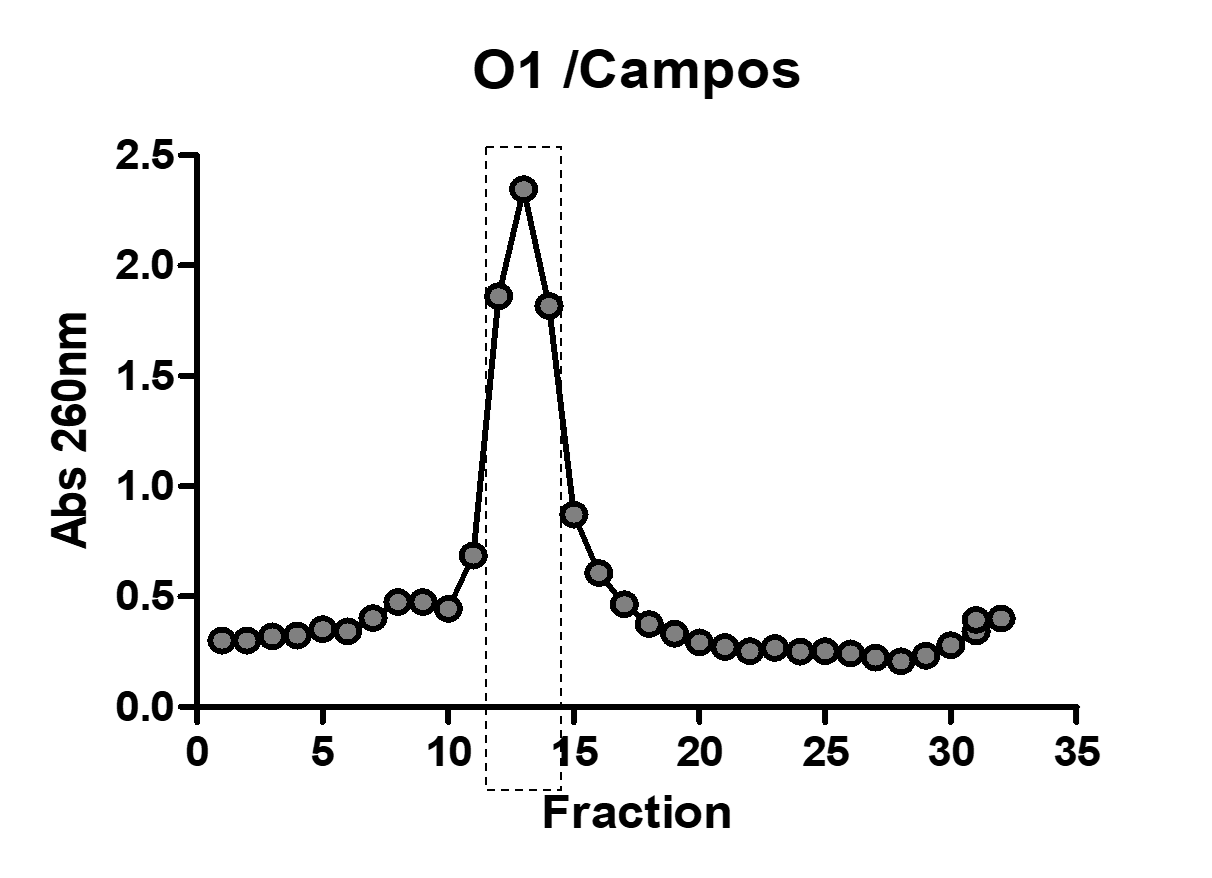

Supplement: S1 Fig — The dotted square indicates the fractions used in ELISA. (TIF) [file pone.0232782.s001.tif]
